# The Rules of Attraction in Central Nervous System Myelination

**DOI:** 10.3389/fncel.2018.00367

**Published:** 2018-10-15

**Authors:** Rafael Góis Almeida

**Affiliations:** Centre for Discovery Brain Sciences, The University of Edinburgh, Edinburgh, United Kingdom

**Keywords:** axon-glia interactions, oligodendrocyte, myelin, attraction, targeting

## Abstract

The wrapping of myelin around axons is crucial for the development and function of the central nervous system (CNS) of vertebrates, greatly regulating the conduction of action potentials. Oligodendrocytes, the myelinating glia of the CNS, have an intrinsic tendency to wrap myelin around any permissive structure *in vitro*, but *in vivo*, myelin is targeted with remarkable specificity only to certain axons. Despite the importance of myelination, the mechanisms by which oligodendrocytes navigate a complex milieu that includes many types of cells and their cellular projections and select only certain axons for myelination remains incompletely understood. In this Mini-review, I highlight recent studies that shed light on the molecular and cellular rules governing CNS myelin targeting.

## Introduction

Vertebrates require myelin, specialized membrane wrapped by oligodendrocytes around axons, for their nervous systems to function. Myelination drastically changes an axon’s physiology: by insulating it and clustering sodium channels in the short unmyelinated nodes of Ranvier, myelin accelerates action potential propagation in a space-efficient manner (Waxman, 1997; Hartline and Colman, 2007), and facilitates high-frequency firing (Fields, 2008; Perge et al., 2012; Sinclair et al., 2017). Oligodendrocytes are now also thought to transfer glycolytic substrates to the underlying axon (Saab et al., 2016; Saab and Nave, 2017). The importance of myelinating oligodendrocytes is underscored by the severe consequences of their disruption in several neurodegenerative conditions (Philips and Rothstein, 2014; Pouwels et al., 2014; Franklin and Ffrench-Constant, 2017). Furthermore, dynamic regulation of myelin is increasingly implicated in cognitive processes including learning, memory, and social interaction (Fields, 2008, 2015; Zeidán-Chuliá et al., 2014; Filley and Fields, 2016). Given how myelin drastically affects neuronal function, it must be targeted precisely to those axons that need it and excluded from incorrect targets. Indeed, not every axon in the central nervous system (CNS) becomes myelinated: for instance, small axons (< 0.2 μm diameter) remain unmyelinated, as do many large axons even in myelin-rich white matter tracts (Olivares et al., 2001; Saliani et al., 2017). Furthermore, neuronal somas and dendrites remain unmyelinated, as do non-neuronal cells. **How is this selectivity achieved *in vivo*, in the complex milieu of the CNS?** The precision of myelin targeting is especially remarkable given that oligodendrocytes cultured in the complete absence of axons readily deposit myelin on inert surfaces (Rosenberg et al., 2008; Aggarwal et al., 2011; Lee et al., 2012; Bechler et al., 2015). Thus, *in vivo*, the tendency of oligodendrocytes to myelinate promiscuously appears tightly regulated by the attraction or repulsion of prospective targets, which we are only now beginning to understand. In this Mini-Review, I will highlight recent studies that shed light on some of the molecular and cellular mechanisms of CNS myelin targeting.

## When Does an Oligodendrocyte Select its Targets?

**FIGURE 1:**
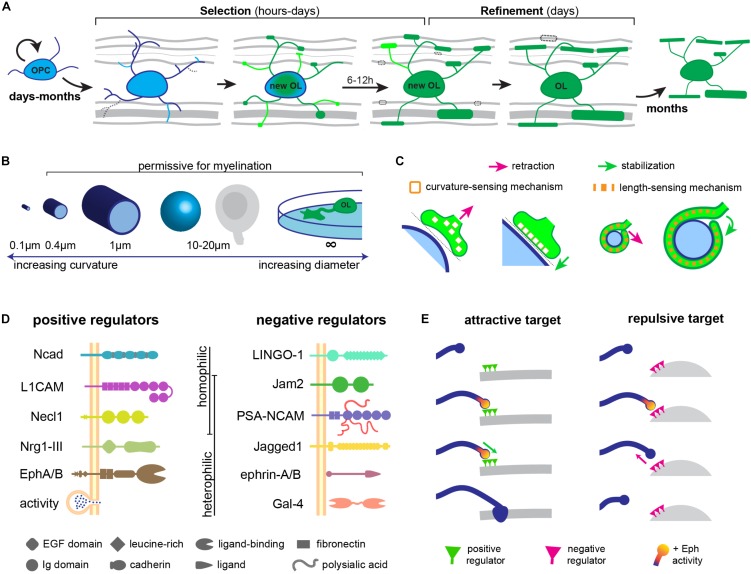
Regulation of CNS myelin targeting. **(A)** myelin is targeted in a short period in the life of an oligodendrocyte (OL): a selection stage during differentiation and a refinement stage after differentiation. **(B)** Biophysical properties of diameter and/or curvature determine the permissiveness of a target for myelination. **(C)** Curvature or length-sensing proteins in oligodendrocyte processes may sense appropriately sized targets. **(D)** Attractive and repulsive cell-adhesion molecules on prospective targets regulating CNS myelination. **(E)** Similar to synapse formation, the temporal dynamics of key signaling pathways (e.g., Eph) may determine the fate of OPC processes during myelin target selection.

Recent studies have now shown that oligodendrogenesis and myelination are life-long processes (Miller et al., 2012; Hill et al., 2018; Hughes et al., 2018), and that myelination in adults can be responsive, for instance, to neuronal activity (Almeida and Lyons, 2017; Mount and Monje, 2017; Sampaio-Baptista and Johansen-Berg, 2017). Thus, myelin targeting mechanisms must operate not just during development, but throughout life. But when does targeting occur during the life-course of an oligodendrocyte-lineage cell? Oligodendrocytes are generated from proliferative oligodendrocyte precursor cells (OPCs), which arise from ventricular germinal zones of the brain and spinal cord and migrate to populate the entire CNS (Richardson et al., 2006). Extrinsic signals and an intrinsic developmental program cooperate to induce OPCs to differentiate into oligodendrocytes, ensheathe axons, and synthesize myelin (Zuchero and Barres, 2013). During and after migration, OPCs and newly differentiated oligodendrocytes extend and retract numerous dynamic filopodia-like processes (Kirby et al., 2006; Hughes et al., 2013) that contact the surrounding milieu, including structures such as axonal and dendritic surfaces, neuronal and glial somas, and blood vessels. Time-lapse imaging studies have provided the remarkable insight that each newly differentiated oligodendrocyte forms all its myelin sheaths in a short hours-long period (Watkins et al., 2008; Czopka et al., 2013). Following this period, stabilized sheaths elongate, but no new sheaths are formed, and only a minority are retracted over the next few days. Importantly, even during the short period of sheath initiation, only a minority of nascent sheaths are retracted (Czopka et al., 2013; Liu et al., 2013), suggesting that most axonal targets are successfully selected beforehand. Thus, the dynamic behavior of filopodia-like processes of newly differentiated oligodendrocytes may well serve to ‘interview’ prospective targets in its vicinity, and local interactions (e.g., between axonal and oligodendrocyte cell-adhesion molecules) may be transduced into the process to stabilize some contacts and retract others. The observation that some newly formed myelin sheaths are subsequently retracted suggests that some correction of mistargeted sheaths that escaped initial selection occurs later. Following differentiation, oligodendrocytes and their myelin are remarkably stable for many months (Tripathi et al., 2017; Hill et al., 2018; Hughes et al., 2018). Thus, myelin targeting occurs in a short period in the life of an oligodendrocyte, and appears to include two stages: first, target *selection* takes place as an oligodendrocyte-lineage cell settles, differentiates, and forms nascent sheaths; followed by a target *refinement* stage in the following days, when some sheaths are retracted (**Figure 1A**).

## Which Signals Target Myelin to Axons?

The contact and recognition of target surfaces by oligodendrocyte processes constitute the first step in CNS myelin targeting. Remarkably, the biophysical properties of a surface as well as the molecules it bears can determine its myelination fate.

### Biophysical Factors

*In vitro*, oligodendrocytes avoid small-diameter fibers under a threshold diameter of 0.4 μm, and myelinate only larger fibers (Lee et al., 2012; Bechler et al., 2015), which is reminiscent of the distribution of myelinated CNS axons *in vivo* (Remahl and Hildebrand, 1982; Hildebrand et al., 1993). Thus, small-diameter cylindrical structures, which present a high-curvature surface to the oligodendrocyte process, appear non-permissive for myelination, in contrast to larger-diameter cylinders with a lower curvature. Oligodendrocytes are also able to target their myelin to non-cylindrical structures *in vitro* such as spherical polystyrene beads around 20 μm in diameter (Redmond et al., 2016), flat surfaces such as glass coverslips (Aggarwal et al., 2011), and conical micropillars (Mei et al., 2014), with an even lower curvature, suggesting that this is an important biophysical constraint for myelination (**Figure 1B**). Do oligodendrocyte processes actively sense curvature? Bechler et al. (2015, 2018) propose that as they myelinate their targets, oligodendrocytes must sense axonal curvature, since they adjust sheath lengths to the axonal diameter. This could rely, for instance, on the curvature-dependent activity of certain membrane-anchored proteins (Chang-Ileto et al., 2011; McMahon and Boucrot, 2015; **Figure 1C**), or on a mechanism analogous to the curvature-sensitive septin cytoskeleton (Bridges et al., 2016; Osso and Chan, 2017). Alternatively, oligodendrocytes could detect the length of the first complete wrap around the axonal perimeter and induce process retraction if under a threshold length (Osso and Chan, 2017; **Figure 1C**).

These *in vitro* observations suggest that biophysical properties of prospective targets, such as their size, could dictate their permissiveness for myelination. In fact, driving an increase in the diameter of cerebellar granule cell axons, which are typically small and unmyelinated, is sufficient to target them for myelination (Goebbels et al., 2017). It remains possible, however, that axonal signals that tightly correlate with axonal diameter could underlie such an effect *in vivo*. Indeed, in the peripheral nervous system (PNS), only axons strictly above 1 μm in diameter become myelinated by Schwann cells, and only these large axons expressed a high level of membrane-anchored Neuregulin 1 (Nrg1) type III (Taveggia et al., 2005). This signal turned out to mediate the correlation of axonal caliber with PNS myelination: genetic ablation of Nrg1-III greatly reduces myelination including in large, supra-threshold axons; while its overexpression in small-diameter, typically unmyelinated axons is capable of converting them to a myelinated fate (Michailov et al., 2004; Taveggia et al., 2005). Thus, similar membrane-anchored axonal signals might mediate the preference of oligodendrocytes for larger targets in the CNS. Indeed, the geometric permissivity of a prospective target cannot fully explain CNS myelin targeting: axons between 0.4 and 1 μm in diameter can be either unmyelinated or myelinated (Hildebrand et al., 1993), and many somas and dendrites of permissive geometries and accessible to oligodendrocytes remain essentially unmyelinated. This indicates that an additional layer of regulation of myelin targeting must exist *in vivo*. In fact, both attractive and repulsive molecular cues can directly regulate myelin targeting (**Figure 1D**).

### Attractive Signals

In the PNS, axonal expression of Nrg1-III is sufficient to instruct Schwann cells to myelinate axons (Michailov et al., 2004; Taveggia et al., 2005). To date, no such molecule has been identified whose disruption completely prevents CNS myelination, suggesting that multiple redundant signals might exist. Indeed, several axonal molecules have been found to promote CNS myelination: although not strictly required for CNS myelination, pan-neuronal overexpression of Nrg1 type III increases the number of axons targeted for myelination in the optic nerve and cortex, including typically unmyelinated small-diameter axons (Brinkmann et al., 2008). Axons and oligodendrocytes also express multiple Eph tyrosine kinase receptors and their ephrin membrane-bound ligands, whose *trans*-interactions induce bidirectional signaling to regulate adhesion in adjacent cells. Primary rat OPCs cultured on EphA4/B1 substrates have increased myelination, suggesting that binding of axonal EphA4/B1 receptors to the oligodendrocyte ligand ephrin-B induces reverse signaling in oligodendrocytes that promotes myelin sheath formation (Linneberg et al., 2015), and could thus target myelin to specific axons. Axonal L1-CAM can bind to contactin and integrin complexes in oligodendrocytes and increase myelination *in vitro* (Laursen et al., 2009). N-cadherin, a calcium-dependent cell-adhesion molecule expressed in both axons and oligodendrocytes, is necessary during the initial axon-OPC interactions, as blocking its function greatly reduced the duration of these contacts, leading to reduced myelination (Schnädelbach et al., 2001; Chen et al., 2017). Genetic ablation of Nectin-like 1, a neuronal member of the synaptic cell adhesion molecule family, reduced the number of spinal cord and optic nerve axons targeted for myelination, albeit only transiently (Park et al., 2008).

An exciting finding is the recent observation that neuronal activity can also regulate the targeting of myelin to specific axons, in addition to long-standing observations that activity also influences OPC proliferation (Barres and Raff, 1993; Li et al., 2010; Gibson et al., 2014), oligodendrocyte differentiation and survival (Hill et al., 2014; McKenzie et al., 2014; Hughes et al., 2018) and myelin formation itself (Demerens et al., 1996; Makinodan et al., 2012; Liu et al., 2012). Recent studies in the zebrafish spinal cord have shown that blocking action potentials or activity-dependent vesicle release disrupted the targeting of myelin to a stereotyped subset of spinal cord axons and the degree of their myelination (Hines et al., 2015; Mensch et al., 2015). Indeed, Hines et al. (2015) observed that synaptophysin-containing vesicles accumulated in the axon at sites of oligodendrocyte ensheathment, suggesting that vesicle cargos such as neurotransmitters or cell-adhesion molecules could attract or stabilize nascent sheaths. In line with these studies, chemogenetic activation of individual somatosensory neurons in mice increased their targeting for myelination (Mitew et al., 2018). *In vitro* studies have suggested that glutamate release, which can locally stimulate oligodendrocyte Fyn kinase signaling and myelin protein synthesis (Wake et al., 2011, 2015), is the key vesicle cargo mediating these effects (Spitzer et al., 2016). However, the precise glutamate receptors in oligodendrocytes that specifically mediate a myelination effect *in vivo* remain unclear (De Biase et al., 2011; Guo et al., 2012), and other signals may play a role, including neurotrophins and cell-adhesion molecules such as Nrg1 (Liu et al., 2011; Lundgaard et al., 2013) and N-cadherin (Tan et al., 2010) whose expression or surface localization are sensitive to activity. Furthermore, Koudelka et al. (2016) found that such activity-regulated myelin targeting is a property of some neuronal subtypes but not others, suggesting that activity-dependent and independent mechanisms cooperate to appropriate myelinate the CNS.

### Repulsive Signals

Given the promiscuity of oligodendrocyte myelination, vertebrates might employ repulsive signals not only to decide which axons remain unmyelinated, but also to temporally control the onset of myelination along axons fated for myelination, and to prevent ectopic myelination of inappropriate compartments within a neuron such as its soma or dendrites. Negative signals may also be employed within individual axons to target myelin to specific domains: recent imaging studies have shown that many CNS axons are only partially myelinated *in vivo*, with long segments remaining persistently unmyelinated, which may be important to modulate their conduction and functional output (Tomassy et al., 2014; Auer et al., 2018; Hill et al., 2018; Hughes et al., 2018).

Negative regulators include some of the Eph-ephrin molecules expressed in axons and oligodendrocyte-lineage cells. Axonal ephrin-A1/B2 *forward* signaling through EphA/B receptors on oligodendrocytes can induce process retraction and reduce myelination *in vitro* and *in vivo* (Linneberg et al., 2015; Harboe et al., 2018). Indeed, ephrin-A1 expression in neurons reduced their myelination in the zebrafish spinal cord, by interacting with the EphA4 receptor on oligodendrocytes, whose knockdown increased the number of axons myelinated by individual oligodendrocytes (Harboe et al., 2018). Ephrin-Eph binding induces both forward signaling in the receptor-expressing cell and reverse signaling in the ephrin-expressing cell which can be regulated independently (Pasquale, 2008). This may explain why an EphA4 substrate can also promote myelination of rat OPCs *in vitro* (Linneberg et al., 2015): due to oligodendroglial ephrin (not Eph)-induced signaling. Both neurons and oligodendrocytes express multiple ephrin ligands and Eph receptors, and the cellular context in which specific ephrin-Eph binding occurs may influence the resulting cell behavior. Thus, a precise combinatorial code of axonal Eph and ephrin molecules could determine myelin targeting in the CNS. Importantly, dysregulation of Eph-ephrin did not affect oligodendrocyte differentiation, suggesting a specific role in regulating cell adhesion and targeting.

Other repulsive signals include polysialylated neural cell-adhesion molecule (PSA-NCAM), which is downregulated at the onset of myelination *in vivo* (Nait Oumesmar et al., 1995; Charles et al., 2000). Blocking PSA-NCAM increased the number of myelinated axons, while overexpressing it reduced myelination *in vivo* (Meyer-Franke et al., 1999; Charles et al., 2000; Fewou et al., 2007), potentially by preventing adhesive interactions. LINGO-1 is a transmembrane protein expressed in both axons and oligodendrocytes (Jepson et al., 2012) whose knock-out (Mi et al., 2005) or transgenic overexpression in neurons (Lee et al., 2007), respectively, increase and decrease the number of axons targeted for myelination in the spinal cord. Jagged1, the transmembrane ligand for the Notch1 receptor expressed in oligodendrocytes, is expressed by retinal ganglion cells in the optic nerve, where it signals to prevent OPC differentiation and myelination (Wang et al., 1998). In Notch1 heterozygotes, more axons become myelinated in the optic nerve, and axons in the molecular layer of the cerebellum, which never become myelinated, are also selected for myelination (Givogri et al., 2002).

Redmond et al. (2016) recently identified a negative regulator of neuronal somatodendritic myelination, the cell-adhesion molecule Jam2. Having determined that oligodendrocytes could myelinate soma-sized polystyrene beads *in vitro*, Redmond et al. (2016) searched for repulsive signals that prevent somatodendritic myelination *in vivo* using an elegant strategy of differential RNAseq profiling. By comparing spinal cord neuron cultures, which should employ such repulsive signals to prevent myelination of their many dendrites and soma, to dendrite-less dorsal root ganglion neuron cultures, Redmond et al. (2016) identified Jam2 as an inhibitory signal. This transmembrane protein with extracellular immunoglobulin domains was able to repel oligodendrocyte processes and reduce myelination *in vitro*, without affecting oligodendrocyte differentiation. Oligodendrocytes were able to myelinate the soma and dendrites of cocultured Jam2-knockout neurons, and Redmond et al. (2016) also find ectopically myelinated neurons in the dorsal spinal cord of the Jam2 knockout mouse. Interestingly, all are Pax2^+^ inhibitory interneurons, suggesting that other neuron subtypes use other signals to prevent somatodendritic myelination (Redmond et al., 2016).

More recently, expression of Galectin-4, a lectin transmembrane receptor for glycoproteins, was observed to be inversely correlated with myelination in the rat brain, and to repel myelin formation *in vitro* (Stancic et al., 2012; Díez-Revuelta et al., 2017). Interestingly, Galectin-4 was observed to localize only to unmyelinated segments in hippocampal and cortical neurons (Velasco et al., 2013; Díez-Revuelta et al., 2017), suggesting that it may be one of the signals responsible for maintaining a ‘patchy’ pattern of myelination along individual axons, patterns which are robustly maintained even upon remyelination (Auer et al., 2018). It will be important to disentangle a ‘direct’ effect of these molecules in regulating myelin targeting and adhesion from a secondary effect on oligodendrocyte differentiation.

## How are Targeting Signals Transduced Into Myelinating Behavior?

The answer is likely to depend on whether they act during target selection, which requires stabilizing or retracting a small, dynamic OPC process; or during refinement, requiring the growth or breakdown of a nascent sheath. It seems plausible that surfaces with a non-permissive geometry, or bearing repulsive signals (e.g., Jam2 or Ephrins) destabilize even the earliest interactions with OPC processes during target selection. How might these signals cause an OPC process to retract? Binding of ephrin-A1 to EphA4 in oligodendrocytes, for instance, induces its phosphorylation and activates an ephexyn1-RhoA-Rock signaling cascade necessary for process retraction (Harboe et al., 2018). Thus, regulation of actin cytoskeleton dynamics, which have independently been shown to underlie the wrapping process (Nawaz et al., 2015; Zuchero et al., 2015), is a likely mediator. Remarkably, this sequence of signaling events is similar to EphA4-dependent signaling underlying dendritic spine retraction (Fu et al., 2007) and axonal growth cone collapse/repulsion (Sahin et al., 2005) in mammalian neurons. This suggests that lessons about myelin targeting may be learned from dendritic and axonal interactions during synaptogenesis, a resemblance we had noted before (Almeida and Lyons, 2014). For instance, a recent study showed that dendritic filopodia of cortical neurons select or reject axonal contacts for synaptogenesis based simply on the kinetics of EphB2 signaling, whereby transient activation leads to filopodia retraction while sustained activation predicts stabilization and synaptogenesis (Mao et al., 2018). It will be interesting to determine if Eph signaling kinetics in OPC processes also predict target selection or rejection (**Figure 1E**).

Other signals are more likely to act during target refinement. For instance, activity-dependent vesicle release may stabilize, rather than attract, nascent sheaths. Neurotransmitter or neurotrophin vesicle cargoes are shared by broad classes of neurons and seem unlikely to regulate target selection on an axon-by-axon basis. Instead, differences in the distribution, timing or frequency of vesicle release in different axons could bias the stabilization and growth of nascent sheaths. How might this be translated to the oligodendrocyte? Two recent elegant *in vivo* studies implicated the second messenger calcium. By imaging the genetically encoded calcium indicator GCaMP6 in oligodendrocytes, Baraban et al. (2018) identified high-amplitude, long-duration calcium elevations within sheaths that eventually retracted, and implicated calpain in the retraction. Similar calcium signatures and calpain involvement regulate dendrite retraction in Drosophila sensory neurons (Kanamori et al., 2013), in another instance of similarity with CNS synaptogenesis. Baraban et al. (2018) also found that among the stabilized sheaths, a higher frequency of low-amplitude, short-duration intracellular calcium elevations predicted faster growth. This was supported by an independent study by Krasnow et al. (2018) who further found that blocking these calcium transients prevented sheath growth, proving that calcium activity is causally linked to myelin elongation. It will be important to determine which effectors lie downstream of calcium for sheath growth. One possibility is that it regulates coordinated cycles of actin assembly and disassembly, implicated in myelin sheath growth (Nawaz et al., 2015; Zuchero et al., 2015). Additionally, targeting signals (transduced by calcium-dependent or independent mechanisms) may converge in well-characterized signaling cascades known to regulate myelination. It will be interesting to assess the activation state of the Akt/mTOR and Erk1/2 pathways within oligodendrocytes during targeting, since they have been implicated in the regulation of wrapping (Goebbels et al., 2010; Domènech-Estévez et al., 2016; Mathews and Appel, 2016) and myelin sheath growth (Ishii et al., 2012, 2013; Fyffe-Maricich et al., 2013; Jeffries et al., 2016). Indeed, hyperactivation of Akt signaling induces hypermyelination in the CNS and PNS, including of typically unmyelinated targets (Flores et al., 2008; Goebbels et al., 2010; Almeida et al., 2018).

## Is There a Hierarchy of Myelin Targeting?

We recently tested whether *in vivo* targeting mechanisms were efficient even in conditions with an excess of myelin, such as in the context of potential remyelinating therapies (Almeida et al., 2018). We analyzed zebrafish mutants with fewer axonal targets for myelination, but normal oligodendrocyte number (Almeida and Lyons, 2016), zebrafish treated with small-molecule epigenetic regulators with increased oligodendrocyte number (Early et al., 2018), and zebrafish and mice with increased myelin production due to stimulation of the Akt1 pathway in oligodendrocytes. Remarkably, despite there being no disruption to any specific molecular targeting mechanism, we observed that myelin was ectopically targeted to neuron somas in the developing spinal cord in all three scenarios. This suggests that some neuron somas may lack strong inhibitory signals and are not normally myelinated due to the finely regulated balance of axonal demand to myelin supply. Interestingly, even in wildtype animals, we found a small degree of myelin targeted to neuron somas. Longitudinal imaging revealed that ectopic myelin is made during the same short period of sheath formation by individual oligodendrocytes, and is corrected over the course of a few days – although not sufficiently in animals with excess oligodendrocytes (Almeida et al., 2018). This suggests that slightly overproducing myelin during development may help ensure robust myelination of appropriate targets, and lends support to the existence of a refinement stage during targeting. Furthermore, in animals with excessive oligodendrocytes, axons that are not normally target for myelination (both of small and large, permissive diameters) remained unmyelinated, despite being accessible to oligodendrocytes. Our observations suggest that myelin may be targeted in a hierarchical manner: first to attractive axons; then to less attractive (but not refractory) targets including some axons and some neuronal somas, and finally excluded from refractory small-diameter or repellent axons. Our study suggests that the long-standing observation that axons can regulate the development of the oligodendrocyte population serves not only to guarantee sufficient myelination of the appropriate targets (Barres and Raff, 1999; Klingseisen and Lyons, 2018), but also to minimize ectopic myelination of permissive structures. Such an additional regulatory layer of myelin targeting, influencing myelination fate by indirect regulation of oligodendrocyte number in a given region, cooperates with the more direct, target-specific regulation of adhesion to ensure the fidelity of CNS myelination.

## Conclusion

The complete repertoire of biophysical, attractive and repulsive factors that regulate CNS myelin targeting is likely larger than the current picture, and the rules by which they govern myelin attraction in the CNS complex. For instance, which molecules prevent ectopic myelination of neuron somas, glial cells, and the vasculature? How do complex geometries, such as those of somas bearing numerous dendrites, influence myelin targeting? How is myelin targeting affected by mechanical forces, or by an oligodendrocyte’s myelinating capacity? How do biophysical factors interact with molecular signals? Some axons smaller than 0.2 μm in diameter actually become myelinated in the mammalian CNS (Bishop and Smith, 1964; Adinolfi and Pappas, 1968; Matthews, 1968) – these axons may need to employ additional attractive signals to improve adhesion to OPC processes (Simons and Lyons, 2013). Are the same targeting mechanisms employed during early developmental myelination, activity-responsive myelination, and remyelination? Future studies may be informed by the coincident disruption or overexpression of multiple signals in individual cells to investigate how targeting mechanisms cooperate to culminate in the precisely myelinated vertebrate CNS.

## Author Contributions

RA conceived and wrote the manuscript.

## Conflict of Interest Statement

The author declares that the research was conducted in the absence of any commercial or financial relationships that could be construed as a potential conflict of interest.
